# Path Optimization of Medical Waste Transport Routes in the Emergent Public Health Event of COVID-19: A Hybrid Optimization Algorithm Based on the Immune–Ant Colony Algorithm

**DOI:** 10.3390/ijerph17165831

**Published:** 2020-08-12

**Authors:** Ziyuan Liu, Zhi Li, Weiming Chen, Yunpu Zhao, Hanxun Yue, Zhenzhen Wu

**Affiliations:** 1 School of Economics and Management, China University of Geosciences, Wuhan 430074, China; liuziyuan@cug.edu.cn; 2 School of Geography and Information Engineering, China University of Geosciences, Wuhan 430074, China; cnlizhi@cug.edu.cn; 3 Faculty of Engineering, China University of Geosciences, Wuhan 430074, China; 20171000703@cug.edu.cn; 4 School of Computer Science, Wuhan University, Wuhan 430072, China; 2018302060341@whu.edu.cn; 5 School of Cyber Science and Engineering, Wuhan University, Wuhan 430072, China; 2018302180071@whu.edu.cn

**Keywords:** transit storage, ant colony algorithm, immune tabu search algorithm, path optimization, medical waste

## Abstract

In response to the emergent public health event of COVID-19, the efficiency of transport of medical waste from hospitals to disposal stations is a worthwhile issue to study. In this paper, based on the actual situation of COVID-19 and environmental impact assessment guidelines, an immune algorithm is used to establish a location model of urban medical waste storage sites. In view of the selection of temporary storage stations and realistic transportation demand, an efficiency-of-transport model of medical waste between hospitals and temporary storage stations is established by using an ant colony–tabu hybrid algorithm. In order to specify such status, Wuhan city in Hubei Province, China—considered the first city to suffer from COVID-19—was chosen as an example of verification; the two-level model and the immune algorithm–ant colony optimization–tabu search (IA–ACO–TS) algorithm were used for simulation and testing, which achieved good verification. To a certain extent, the model and the algorithm are proposed to solve the problem of medical waste disposal, based on transit temporary storage stations, which we are convinced will have far-reaching significance for China and other countries to dispatch medical waste in response to such public health emergencies.

## 1. Introduction

Medical waste refers to wastes produced by medical and health institutions in the course of medical treatments, preventions, health care, and other related activities, which are directly or indirectly infectious, toxic, and have other hazardous characteristics. For large and medium-sized cities, how to set up an effective medical waste transport system has long been the focus of the world. The academic community has also put forward many ideas and solutions. For example, Gerasimos Mantzaras [1] developed an optimization model to minimize the cost of a collection, haul, transport, treatment and disposal system for infectious medical waste (IMW). Qiu Cheng [2] believed that a BOT (build–operate–transport) model could be used to solve the problem of medical waste treatment, and selected Kunming City as an example for the research. Liu Xiaoli [3] analyzed the current situation of medical waste transport in primary hospitals with Wuhan as an example, and believed that communication and cooperation between multiple departments should be strengthened, appropriate managing models should be explored, and we should attach more attention to training and supervision. Oweis, R. [4] researched the medical waste transport system of King Hussein Medical Center in Jordan and found that there were a lot of gaps that needed to be resolved in the future, including effective isolation, use of coding and colored bags, better handling and transport methods, better monitoring and tracking technology, and the need for training and an awareness plan for people. Kumar, A. [5] considered the transportation of medical waste as a key step in its management. Regular training programs for all sectors of health care workers are needed, with a special focus on waste handlers. With the development of the information age, artificial algorithms have been widely used to all kinds of fields, including the public opinion polarization process [6,7,8,9], fall detection [10], analysis of user satisfaction [11], welding flame detection [12], and road networks [13,14].

Through the previous research, it was found that one of the key directions to solving the problem of urban medical waste transport is to establish a temporary storage station and further build an efficient hospital–temporary storage station transport model. By doing the analysis, it is believed that this is a typical Vehicle Routing Problem (VRP), which could be solved by studying vehicle routing problems with load constraints (CVRPs), an important branch of VRP. As an extension of VRP, CVRP, which is a typical Nondeterministic Polynomially(NP) Problem, has become a hotspot in the field of operation research and combinatorial optimization.

VRP is the abbreviation of the vehicle routing problem. The problem is that there are N cars, all starting from the origin; each car visits some points and then returns to the origin, requiring all points to be visited, seeking the shortest driving distance or the minimum number of vehicles required or the minimum long-driving distance. CVRP refers to VRP with capacity limitations.

The NP problem is a complex problem where it cannot be determined whether the answer is found in polynomial time, but it can be verified if the answer is correct in polynomial time.

At present, many researchers from all over the world have adopted a large number of heuristic solutions and have proposed a large number of models and extensions for CVRP. For example, Lucía Cazabal-Valencia analyzed CVRP with ellipsoidal distances, which included an inventory model with uniformly distributed demands [15]; F.E. Zulvia proposed a hybrid ant-colony optimization and genetic algorithm for solving CVRP with a time window and fuzzy travel time and demand [16]; Osman Gokalp proposed a novel algorithm based on iterated local search and the random variable neighborhood descent metaheuristic method for the purpose of solving CVRP [17]; Mahmuda Akhtar presented a modified backtracking search algorithm in CVRP models, with the smart bin concept to find the best optimized waste collection path distances [18]; Sami Faiz developed a decision support system for solving CVRP that integrated GIS enriched by a tabu search model [19]; Chengming Qi proposed a two-stage hybrid Ant Colony System (ACS) algorithm for CVRP that minimized the number of vehicles used and travel cost [20]; A. Gomez presented a new artificial bee colony algorithm for solving CVRP [21]; M. Ammi and S. Chikhi proposed an island model for solving CVRP, which consists of using a paradigm, called the island model, that rules the cooperation held by different islands [22]; S.L. Gadegaard proposed a new polynomially sized formulation of the well-known symmetric CVRP [23]; Yiyong Xiao presented a mathematical optimization model to formally characterized the fuel consumption rate considered in CVRP [24]; Rodrigo Linfati proposed a heuristic algorithm for the reoptimization of CVRP in which the number of customers increases, which uses the proposed performance to reduce route dispersion and minimize length [25]; Jiashan Zhang presented a novel two-phase heuristic approach for the CVRP to overcome limitation [26]; Ali Asghar Rahmani Hosseinabadi introduced a new metaheuristic optimization algorithm to solve CVRP that is based on the law of gravity and group interactions [27]; Asma M. Altabeeb proposed a new hybrid firefly algorithm to solve CVRP [28]; Hadi Karimi investigated various stabilization techniques for improving the column generation algorithm and proposed a novel stabilization technique specialized for CVRP [29]; A.K.M. Foysal Ahmed proposed an efficient algorithm, bilayer local search-based particle swarm optimization, along with a novel decoding method to solve CVRP [30]; Mauro Dell’Amico proposed a new iterated local search metaheuristic method for CVRP that also includes a vital mechanism from the adaptive large neighborhood search combined with further intensification through local search [31]; R. Baldacci described a new integer programming formulation for CVRP based on a two-commodity network flow approach [32]; Fernando Afonso Santos introduced a branch-and-cut-and-price algorithm for two-echelon CVRP [33]; Jiafu Tang developed a BEAM–MMAX algorithm that combines a MAX–MIN ant system with beam search to solve CVRP [34]; Jacek Mańdziuk proposed a solution to CVRP with traffic jams, which relies on application of the upper confidence bounds applied to the trees method [35]; Juan Rivera presented a mixed integer linear program and a multistart iterated local search, calling a variable neighborhood descent to solve multitrip cumulative CVRP [36]; Vincent F. Yu presented a symbiotic organism search heuristic method for solving CVRP [37]; Ehsan Teymourian presented an enhanced intelligent water drop and cuckoo search algorithm for solving CVRP [38].

Most of the research mentioned above only focused on the improvements of the CVRP solution and some preliminary applications of the theoretical model without considering practical applications such as the transport of medical waste. Considering the particularity of the transport problem of medical waste, it is of paramount importance to establish a targeted model. In terms of the algorithm, the specific model discussed in this paper not only simplifies CVRP, but also involves location and its allocation; the algorithm above has its limitations, and we need to put forward a specific algorithm to solve this model. Moreover, due to the emergency, urgency, and criticality of the pandemic, the establishment of an efficient transport system model of medical waste has become a priority in response to the emergent public health crash. In this paper, the immune algorithm, the q-value method, and the improved ant colony algorithm are applied to the model to solve the path planning problem of the transport of medical waste. Finally, the strategy under the simulation parameters is given in the simulation experiment. The model could provide a valuable reference for emergency vehicle scheduling and transport of medical waste before and after such unprecedented public health events.

The rest of this paper is organized as follows: In Section 2, a complete mathematical description of the model is proposed. In Section 3, the theory of the immune algorithm and the ant colony algorithm, which are used to lead to our improved algorithm, is explained, in addition to the initialization of some key parameters. In Section 4, Wuhan is taken as an example to verify the algorithm and to solve the model in the actual background. Additionally, Section 5 gives a review and conclusion of the whole work.

## 2. Mathematical Model Establishment

### 2.1. Overview of the Problem

The studies of this paper are to establish a number of transport stations and an efficient medical waste transport model between hospitals and transport stations and, eventually, optimize the transportation paths. This problem is a nonconvex and nonsmooth nonlinear programming problem with complex constraints. It is an NP-hard problem, and it is difficult to solve in a traditional way.

The problem can be divided into the following two subproblems:

The first subproblem is to establish several waste transport stations for numerous existing hospitals. When establishing the model of the transport stations, factors including the environment and traffic need to be considered.

The second subproblem is based on the first subproblem. After establishing the transport stations, we need to optimize the transportation paths between each of the hospitals and the corresponding transport stations. In path optimization, factors such as the load capacity of the transport vehicle and the amount of generated waste are taken into account. The second subproblem is similar to CVRP.

In addition to the two problems above, considering the practical background of this problem, to reasonably initialize the amount of medical waste and the number of transport vehicles is also required. However, these problems are not major. As a result, their solutions are not presented as separate subproblems.

### 2.2. Mathematical Model of the First Problem

To solve the problem of finding suitable transport stations, we have established the following mathematical description:

(1) $$F=min\overset{}{\underset{i\in N}{\sum }}\;\overset{}{\underset{j\in M_{i}}{\sum }}\;\omega_{i}d_{ij}Z_{ij}$$

(2) $$\overset{}{\underset{j\in M_{i}}{\sum }}\;Z_{ij}=1,i\in N$$

(3) $$Z_{ij}\leq h_{j},i\in N,j\in M_{i}$$

(4) $$\overset{}{\underset{j\in M_{i}}{\sum }}\;h_{j}=p$$

(5) $$Z_{ij},h_{j}\in \left\{0,1\right\},i\in N,j\in M_{i}$$

(6) $$d_{ij}\leq s$$

Explanation of the variables used in the model: N=1,2,…n is the serial number set of all hospitals;

s is the upper limit of the distance between the transport station and the hospital we set;

$M_{i}$ is the set of candidate transport stations, with a distance to hospital i less than s;

$\omega_{i}$ represents the amount of medical waste generated by hospital i every day;

$d_{ij}$ represents the distance from hospital i to the nearest transport station j;

$Z_{ij}$ is a Boolean variable, indicating whether there is a transshipment relationship between hospital i and transport station j. When $Z_{ij}$ is 1, it means that the waste of hospital i will be transported to the transport station j;

$h_{j}$ is a Boolean variable. When $h_{j}$ is 1, it indicates that location j is selected as the relay station.

p is the number of transport stations we set, which is a constant.

Explanation of the formula in the model:

Formula (1) is the goal of this model, which ensures a minimum transportation cost from the hospital to the transport station;

Formula (2) ensures that the waste of each hospital will only be transported to the only transport station corresponding to this hospital, which is convenient for management;

Formula (3) ensures that hospital waste can only be transported to the location set as a transport station;

Formula (4) ensures that the number of points selected as a transport station is p;

Formula (5) indicates that Z and h are Boolean variables;

Formula (6) indicates that all transport stations should be within the transportation range of the hospital.

In addition to the establishment of these transport stations, we also need to consider their impact on ecological and human-activity areas. Therefore, on the basis of the mathematical model, we also used the buffer analysis technology of ArcGIS software to exclude the areas where the environmental assessment indicators are unqualified.

This exclusion belongs to the application of the model. Thus, this exclusion is mentioned in Section 3.4.1, later in the article.

### 2.3. Mathematical Model of the Second Problem

In the first problem, we established several transport stations, and each transport station is responsible for a certain number of hospitals. The waste of these hospitals only needs to be transported to their corresponding transport stations.

Hence, in path optimization, considering the transportation between each transport station and the corresponding hospital, as well as the transportation between the hospitals responsible for the same transport station, are needed. Nothing else needs to be considered.

For example, suppose there are two transport stations, A and B. A is responsible for two hospitals, C and D. Then, we merely need to consider the transportation between A, C, and D, not the transportation between A and B, nor the transportation between B, C, and D.

Therefore, the problem has been simplified to a scheduling problem between one transport station and its corresponding hospitals.

After understanding this simplification, we improved CVRP to make it suitable for our problem. Then, we could get the following mathematical model:

(7) $$J=min\overset{m}{\underset{k=1}{\sum }}\;\overset{n}{\underset{i,j=0,i\neq j}{\sum }}\;c_{ij}x_{ij}^{k}$$

(8) $$\overset{n}{\underset{j=1}{\sum }}\;x_{0j}^{k}=\overset{n}{\underset{j=1}{\sum }}\;=1,k\in \left(1,2,\ldots ,m\right)$$

(9) $$\overset{m}{\underset{k=1}{\sum }}\;\overset{n}{\underset{j=0,i\neq j}{\sum }}\;x_{ij}^{k}=1,i\in \left(1,2,\ldots ,m\right)$$

(10) $$\overset{m}{\underset{k=1}{\sum }}\;\overset{n}{\underset{i=0,i\neq j}{\sum }}\;x_{ij}^{k}=1,j\in \left(1,2,\ldots ,n\right)$$

(11) $$\overset{n}{\underset{i=1}{\sum }}\;d_{i}\overset{n}{\underset{j=0,i\neq j}{\sum }}\;x_{ij}^{k}\leq b_{k},k\in \left(1,2,\ldots ,m\right)$$

Explanation of the variables used in the model:

For one transport station, m is the number of transport vehicles we assign to it;

$d_{i}$ is the amount of daily waste generated by hospital i;

$c_{ij}$ is the distance of the transport vehicle from hospital i to hospital j;

$b_{k}$ is the capacity of transport vehicle k;

$x_{ij}^{k}$ is a Boolean variable used to indicate the vehicle’s path. When $x_{ij}^{k}$ is 1, it means that vehicle k visits hospital j immediately after visiting hospital i.

Explanation of the formula in the model:

Formula (7) is the goal of the model, which ensures that the transportation path between each transport station and its corresponding hospitals is the shortest;

Formula (8) indicates that the transport vehicles depart from the transport station and return to the transport station after completing the transportation task. Each car’s path forms a Hamilton tour;

Formulas (9) and (10) indicate that the vehicle must serve all hospitals and they can only be served once;

Formula (11) indicates that when each vehicle serves the hospitals, its own load cannot be lower than the total amount of medical waste of the hospitals it passes through.

## 3. Description and Application of Algorithms

Evolutionary algorithms have strong robustness to solve complex optimization problems [39]. The immune algorithm (IA) is a new intelligent algorithm inspired by biological immune systems. It applies the diversity of the immune system to generate and maintain the diversity of the population, which overcomes the “premature” problems that are difficult to deal with in the general optimization process, especially in the multimodal function, and finally obtains the global optimal solution [40]. The ant colony optimization (ACO) algorithm, as one of the modern heuristic algorithms, has received wide attention since it was proposed. It has the advantages of positive feedback, parallelism, and robustness, which show good performance in solving task allocation and path optimization. However, at the same time, the ant colony algorithm also has some defects, such as when solving large-scale problems, as problems such as long operation time, slow convergence speed, and ease of getting into the local optimal solution frequently happened. The tabu search (TS) algorithm constructs a tabu table to avoid getting into the global optimal solution and improves the optimization ability of the algorithm.

In this paper, a hybrid intelligent algorithm of IA–ACO–TS is proposed to solve the problem of the model in a realistic background. As an extension of the emerging intelligent algorithm and the genetic algorithm, the immune algorithm has good robustness and global search ability for the location of the transport station, the coordinates of the distribution of hospitals, and the transport station are selected.

For the path optimization problem between the transport station and the hospitals, we used the ant colony optimization algorithm and the tabu search algorithm to solve it, which is a vehicle routing problem with load constraints (CVRP). Considering that the ant colony algorithm can easily fall into local optimum and its stability is not good enough, we optimized the pheromone updating mechanism using the basic idea of the tabu search algorithm and set the tabu table and aspiration criterion in order to improve the effectiveness of the algorithm to solve the problem.

### 3.1. Location Using Immune Algorithm

#### 3.1.1. Algorithm Processes

**Figure 1:**
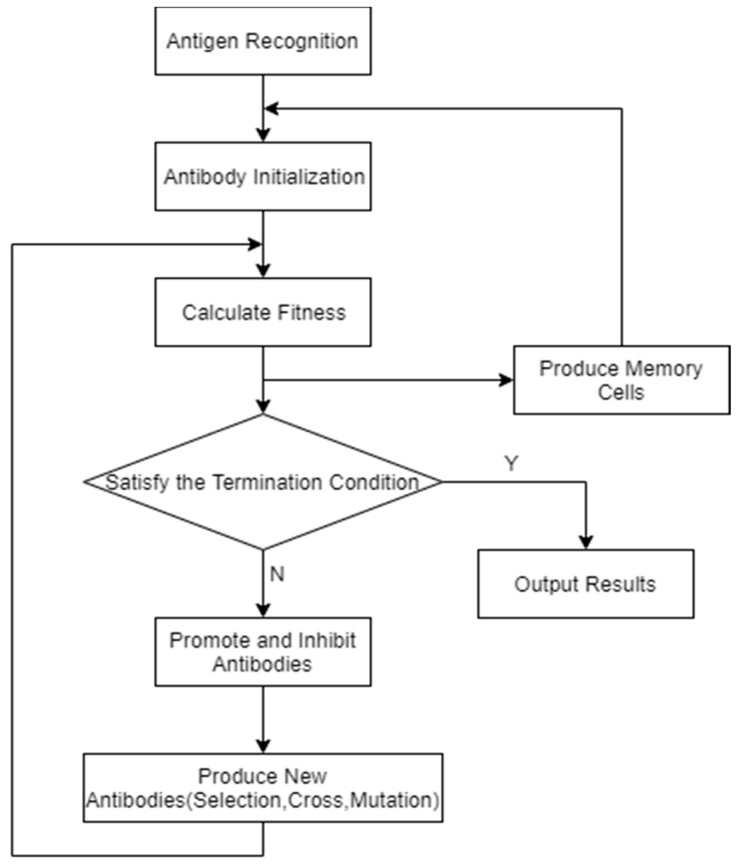
An illustration of the immune algorithm.

The specific process of the immune algorithm is shown in the flowchart in Figure 1. We can find more specific information in [41].

#### 3.1.2. Antibody Initialization

If the memory library is not empty, then the antibody population is generated from the memory library. Otherwise, the initial population would be generated in the feasible solution space randomly. Simple coding is used here. Each selection of the location can produce an antibody of length P (P for the number of transport stations), while each antibody represents the sequence selected for the hospital to which the transport station belongs.

For example, consider the problem containing 10 hospitals, with 1 to 10 being the sequence of hospitals. Suppose you pick three of them, and antibody 1, 2, 3 represent a feasible solution, which means 1, 2, 3 have been chosen for the transport stations.

#### 3.1.3. Evaluation of the Diversity of Solutions

(1) Affinity between Antibody and Antigen 

Antibody–antigen affinity indicates the recognition degree of antigen to antibody. According to this model, we set up affinity function A:

(12) $$A=\frac{1}{\sum_{i\in N}^{}\;\sum_{j\in M_{i}}^{}\;\omega_{i}d_{ij}Z_{ij}-C\sum_{i\in N}^{}\;min\left\{\sum_{j\in M_{i}}^{}\;Z_{ij}-1,0\right\}}$$

The first term of the denominator is the objective function of the model, and the second term gives a penalty function to the solution violating the distance constraints. C is a large, positive number. We use the affinity between the antibody and antigen as a fitness function.

(2) Affinity between Antibodies 

The antibody–antibody affinity reacts to the degree of similarity between antibodies. In this paper, we use the r-continuous bit matching rule proposed by Forrest in 1994 to calculate the affinity.

(13) $$S_{v,s}=\frac{k_{v,s}}{L}$$

where $k_{v,s}$ is the number of the same bits between antibody v and s. L is the length of the antibody. 

(3) Antibody Concentration 

The concentration of the antibody is the percentage of the antibody’s population to the whole population.

(4) Expected Reproductive Probability 

In a population, the expected reproductive of each individual is determined in part by antibody–antigen affinity A and antibody concentration.

(14) $$P=\alpha \frac{A}{\sum A}+\left(1-\alpha \right)\frac{C_{v}}{\sum C_{v}}$$

#### 3.1.4. Immune Operation

(1) Selection. We used the roulette selection mechanism for select operation. The individual selection probability is expected to be the more reproductive probability.
(2) The algorithm uses a single-point crossover for crossover operation.
(3) Mutation random selection of mutation bits is used for mutation operation.

### 3.2. Path Optimization Using Ant Colony Optimization Algorithm and Tabu Search Algorithm

Ant colony optimization (ACO) is an optimization algorithm that simulates the foraging behaviors of ants. It was first proposed by Italian scholar Dorigo, M. and others in 1991 and used in solving TSP (the traveling salesman problem) for the first time. ACO uses the concept of pheromones to simulate the communication mechanism between individuals, which endows the artificial ant with certain memory abilities, so the path chosen would be optimized gradually with the increase of the number of drops. The pheromone update formula for each path is presented as follows:

(15) $$\tau_{ij}\left(t+n\right)=\left(1-\rho \right)\tau_{ij}\left(t\right)+\Delta \tau_{ij}\left(t\right)$$

The formula represents the updating of pheromone on path (i,j) at time (t+n); $\tau_{ij}\left(t\right)$ represents the pheromone on path (i,j) at time t; ρ represents the information volatilization factor.

When the ant colony algorithm is used to solve CVRP, ants produce pheromones on each path. The pheromones indicate the attraction degree of the next customer j to the vehicle k. When the vehicle’s load meets the needs of customers, the vehicle will select customers according to the transport rules we set. After all the selected customers form loops, the pheromone of each path is updated, and then an iteration is operated. When the iteration reaches the maximum number of times, a PARETO solution can be obtained. Compared with other heuristic algorithms, ACO has higher performance.

When ant k is at node i, solve for the probability density function to the next node according to the following rules:

(16) $$p_{ij}^{k}\left(t\right)=\{\frac{\tau_{ij}^{\alpha }\left(t\right)\eta_{ij}^{\beta }\left(t\right)}{\sum_{u\in N_{i}^{k}\left(t\right)}^{}\;\tau_{it}^{\alpha }\left(t\right)\eta_{iu}^{\beta }\left(t\right)},\left(i,j\right)\in N_{i}^{k}\left(t\right)\;0,\left(i,j\right)\notin N_{i}^{k}\left(t\right)$$

In the expression, $\tau_{ij}$ represents the concentration of pheromone on edge (i,j), $\eta_{ij}$ is the attraction of transporting from one node to another, and we could let $\eta_{ij}=\frac{1}{d_{ij}}$, where $d_{ij}$ is the distance between the nodes. $N_{i}^{k}\left(t\right)$ is the set of optional nodes. When the transport probability is worked out, a random number table is generated to determine the next transport station. According to the above description, ACO is applied to solve the vehicle routing problem in a specific region. The steps of the algorithm are as follows:

(1) Parameters Initialization. 

Set the maximum number of iteration $N_{c}$, the current optimal shortest path length $shortest_{len}$, the customer demand load[J], and the current optimal path tabu table $tabu_{min}$. Calculate the pheromone’s initial value $\tau_{0}$, transport attractiveness $\eta_{ij}\left(i,j=1,2,\ldots ,n\right)$. Place M vehicles in the depot and initialize the tabu table ant[k].tabu(k=1,2,…,m), the length of the path ant[k].length(k=1,2…,m), the load on the vehicle ant[k].load(k=1,2…,m), and the pheromone on the path for each vehicle.

(2) Initialize the location of the vehicle.

The vehicles are added to ant[k].tabu and the values of ant[k].length and ant[k].load are updated.

(3) Path Selection.

On the premise of satisfying the vehicle load condition, the vehicle selects the customer according to Formula (2) If the target load is greater than the vehicle’s residual load, the vehicle returns to the depot. Add the select customer or depot to ant[k].tabu and update ant[k].length and ant[k].load.

(4) Local updating of the pheromone. 

Every time a vehicle makes a choice, it updates the path (i,j) that it has just traveled, according to Formula (1).

(5) Global dynamic updating of pheromones. 

The shortest path in the current iteration is counted, and ant[k].length is compared to the shortest. If $ant\left[k\right].length\leq shortest_{len}$, then we replace $shortest_{len}$ with ant[k].length and replace $tabu_{min}$ with ant[k].tabu and the optimal path is updated globally.

### 3.3. Processes of IA–ACO–TS

**Figure 2:**
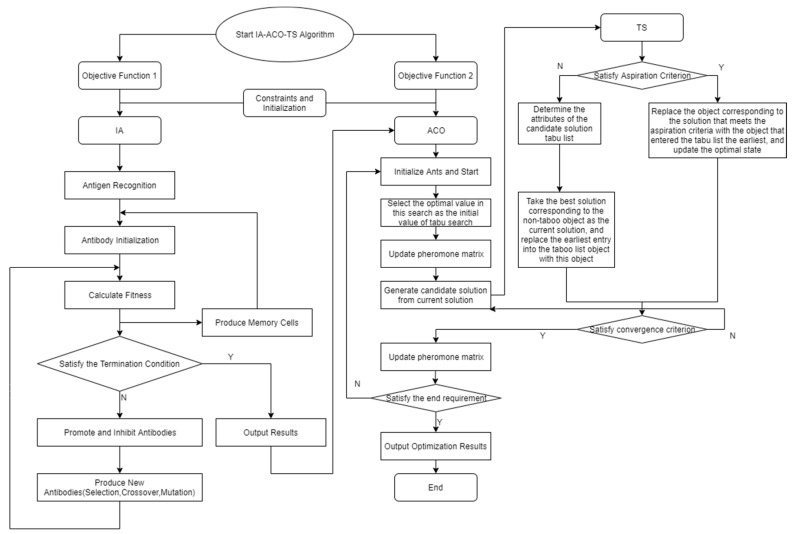
An illustration of the immune algorithm–ant colony optimization–tabu search (IA-ACO-TS) process.

The specific process of the whole algorithm is shown in the flowchart in Figure 2.

### 3.4. Initialization of Parameters and Environment Assessment

#### 3.4.1. Use ArcGIS to Exclude Inappropriate Locations

According to “Technical principles for environmental impact assessment of construction projects of hazardous waste and medical waste disposal facilities (for trial implementation)”, the location of hazardous waste and medical waste disposal facilities must strictly abide by the relevant provisions of national laws, regulations, and standards. Additionally, the site selection should be based on a comprehensive analysis of the social environment, natural environment, site environment, engineering geology, hydrogeology, climate, emergency rescue, and other factors. According to the assessment guidelines of the actual situation in Wuhan, this paper summarizes the exclusion criteria that are not suitable for the construction of medical waste disposal facilities in the analysis process using ArcGIS, as shown in Table 1.

#### 3.4.2. Allocation of Carrying Capacity by *Q*-value Method

Under such epidemics, the distribution of limited carrying capacity is a common resource allocation problem, which is a typical case of the application of mathematics in the political field. The goal is to be as fair and reasonable as possible when a large group allocates certain resources to a small group.

Since carrier forces are quantized, the key to solving the problem is to propose a fairness measure that satisfies the following five principals:

1. An increase in population on one side would not result in the loss of a place on the other;
2. On an average basis throughout the period, each side will receive its own share;
3. Increases in the total number of places would not result in a decrease in the number of places for one side;
4. Neither side’s places will deviate from its proportional places;
5. The absence of a place transport from one side to the other will bring both sides closed to their share.

Since there is no absolute fair distribution, people have devoted themselves to the study of relative fairness. The famous Q-value method was developed in 1982 by D.N. Burghes and I. Huntley, which is a simple method that can overcome some contradictions of other methods, so it has been widely used in the problem of fair allocation of resources.

For the specific problem in this paper, we have the following model:

Suppose that there are m vehicles participating in n distributable transport stations, where the resources of i are $p_{i}$. The total resource of m is $p=\sum_{i=1}^{m}\;p_{i}$, and the vehicles for i are $n_{i}$. How do we find a set of integers $n_{1}\;n_{m}$ that make $\sum_{i=1}^{m}\;n_{i}=N$ as fair as possible?

The ideal fair distribution scheme is the distribution according to the proportion of resources; that is, the number that the i should distribute is $n_{i}=\frac{p_{i}}{p}N,$ which often is not an integer, and a “round-off” leads to unfairness, so the classical Q-value method was put forward.

The Q-value method is used to derive a standard quantity $Q_{i}$ for the allocation of seats, $Q_{i}=\frac{p_{i}^{2}}{n_{i}\left(n_{i}\right)+1}\left(i=1,2,\ldots ,m\right)$. According to it, we determine which side should be allocated the next seat, as follows:

First, each side is assigned a seat based on the calculated value of Qi. The larger side has priority to get the next seat. Then, calculate the value again, and so on, until all seats are allocated.

We applied the Q-value method to solve the problems of assigning buses to each transport station in Wuhan. In this paper, the total quantity of waste in each transport station is taken as the parameter p in the method, and it is known that Wuhan has 50 vehicles that can transport waste at present. The p-value is substituted into the formula, and the total number of vehicles assigned to each transport station is solved circularly.

The Q-value method is used to allocate the vehicles, and the carrying capacity is reasonably distributed in each area of Wuhan so that the existing manpower and material resources can be used more evenly and efficiently.

#### 3.4.3. Initialization of the Amount of Medical Waste Generated by Each Hospital

Based on the data we have, we initialize the amount of medical waste generated by hospitals of all levels. The result is shown in Table 2.

For the amount of medical waste, the number is initialized by statistics. Hospitals are divided into three levels according to the objective hospital grade, with the lowest level being 1 and the remaining two levels being 1.25 and 1.54, respectively. In this model, we consider the temporary shelter hospitals established during the special period of the pandemic. Considering that the shelter hospitals only accept patients with coronavirus and their scale is limited, taking into account the fact that this part of the patients’ household waste would be classified as medical waste, all the shelter hospitals in the second class are clarified.

## 4. Case Analysis

Since the outbreak of COVID-19 in Wuhan in December 2019, the epidemic has escalated rapidly, with more than 400,000 confirmed cases worldwide and a total of 35 countries declaring a state of emergency. As another significant field of epidemic prevention and control, the treatment of highly infectious medical waste has also been attached with great importance by the Chinese government. With the reference of the latest data from the Ministry of Ecology and Environment of the People’s Republic of China, the average daily output of medical waste in Wuhan before the outbreak was 400,000 tons, while after the epidemic developed at an unprecedented level, the topmost output of medical waste was 2.4 million tons, whose rate was much faster than its incineration at medical waste disposal centers.

During the period of epidemic prevention and control, medical waste generated from the medical treatment of confirmed and suspected patients and their close associations, as well as people isolated at home, was highly contagious, and therefore territorial medical waste was completely cleaned every day, which burdened the medical waste disposal units heavily. Since the disposal centers did not have excess disposal capacity, many designated treatment hospitals also needed to concentrate on sanitizing the excessive medical waste immediately.

Although Wuhan’s medical waste treatment capacity has greatly improved, it is still in a “tight balance”, with a daily medical waste loading rate of 93.2 percent, as the director of the Emergency Response Office of the Chinese Ministry of Ecology and Environment said at a press conference. He mentioned that we are now faced with many problems, such as the relative lag of the linkage efficiency of various positions, the inability to transport medical waste in a punctual way, and the high pressure on communities and medical institutions (including 48 designated hospitals and 16 shelter hospitals). The question of how to solve these problems of the existing medical waste management system in public health emergencies is considerable, for further reducing the risk of secondary transmission of the virus, reducing the transportation time cost of medical waste, and promoting the progress of epidemic prevention and control.

Based on the major public health event in Wuhan, the transport efficiency of medical waste is not enough; this paper takes Wuhan during the epidemic period as an example to verify our model. The data of Wuhan is used for data visualization (dataset information, hospital/shelter hospital coordinates and relay station coordinate scatter points, IA–ACO–TS parameter setting, result visualization) to verify the applicability and validity of the model. The research also provides the perspective of medical waste transport for the world to fight against the epidemic.

### 4.1. Elimination of Inappropriate Areas Using ArcGIS

**Figure 3:**
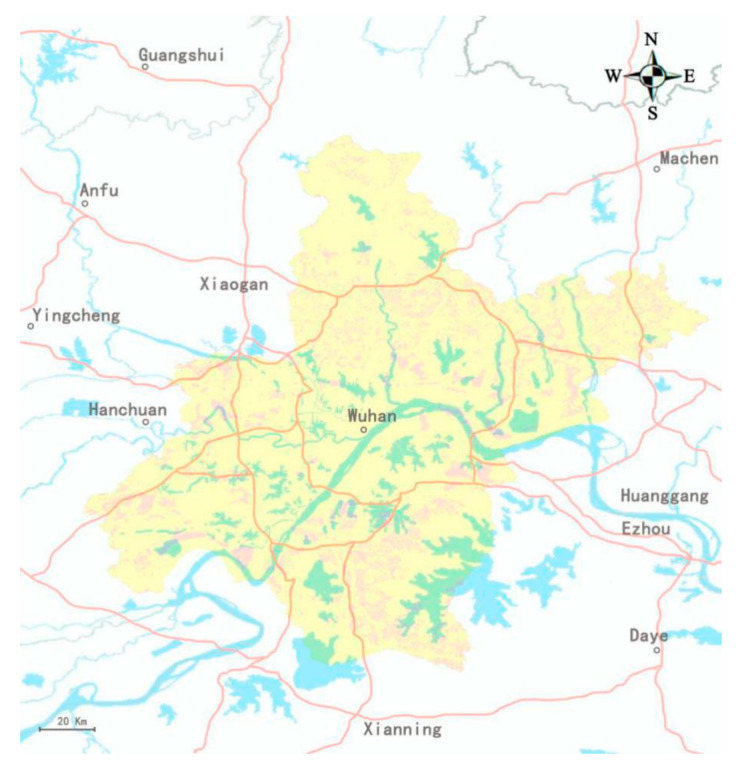
Screening medical waste disposal facility location areas based on ArcGIS software.

The first step in setting up medical waste disposal sites is using the criteria of Table 2 to exclude inappropriate areas based on GIS software. As shown in Figure 3, the yellow areas are unsuitable and the pink areas are suitable for the construction of medical waste disposal facilities.

Given that most of the clinical waste is generated in urban areas, in order to minimize transportation costs, we needed to select a location that is closer to the city center, acting as the final disposal point, the coordinates of which are 30.491111, 114.182922.

### 4.2. Site Allocation Results

**Figure 4:**
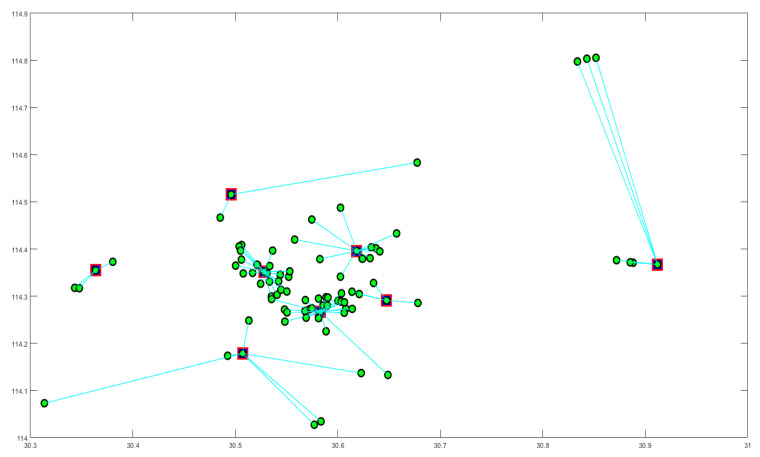
Screening transit points through the quantities of station in Wuhan.

Using the immune algorithm to optimize our calculation, we obtained the geographical coordinates of each relay station and then screened the stations according to the environmental evaluation criteria. The result is shown in Figure 4 and Table 3.

**Figure 5:**
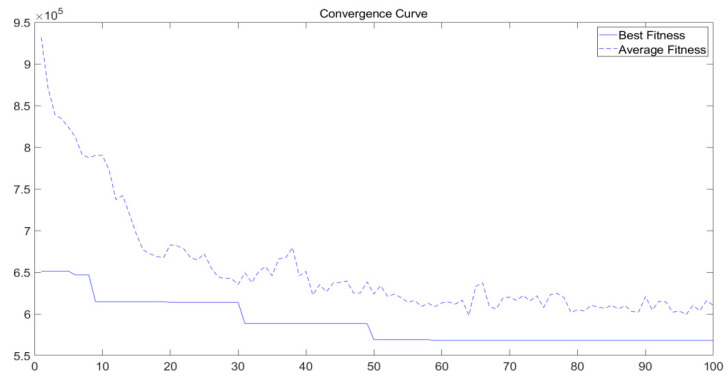
Convergence of the immune algorithm.

As shown in Figure 5, with the increase in the number of iterations, the results gradually converged and we obtained a relatively stable optimal solution.

### 4.3. Optimization of Vehicle Allocation

The Q-value method is used to assign the corresponding vehicles to each transport station. The error function is defined as

(17) $$\varepsilon_{i}=\left|p_{i}-\frac{n_{i}}{\sum_{i\in I}^{}\;n_{i}}\right|$$

where i is the staging area, n is the number of vehicles allocated, and p is the proportion of waste to the total. After calculation, the vehicle allocation and the resulting error are shown in Table 4.

### 4.4. Results of Path Optimization

**Figure 6:**
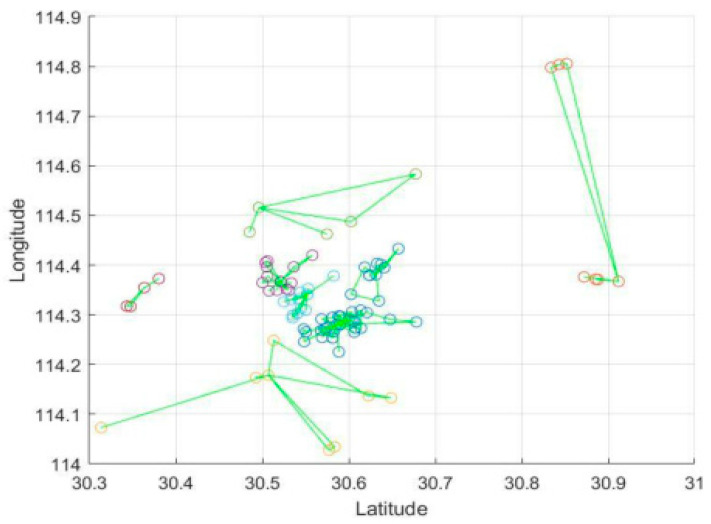
Path planning results for transportation to transit stations in a Wuhan hospital using data modeling.

To demonstrate the effectiveness of our algorithm, we applied real data from Wuhan, including its current capacity and the distribution of hospitals, as well as the shelter hospitals, to the model. The final road map is shown in Figure 6.

As it can be seen, there are eight relay stations in total in the diagram, which are color-coded. Each transport station has its corresponding hospital. The green line is the result of path optimization by utilizing the ant colony–tabu hybrid algorithm. This result ensures the efficiency of medical waste dispatch in the area controlled by each transport station effectively.

**Figure 7:**
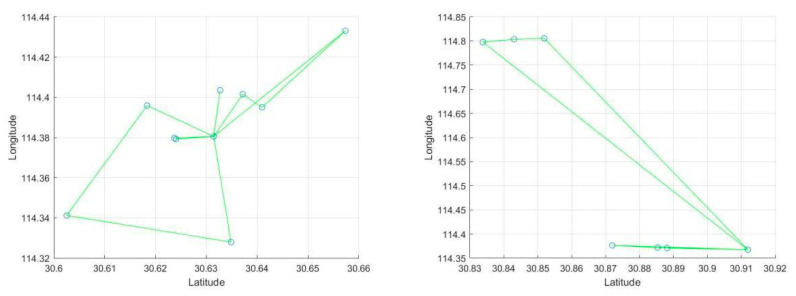

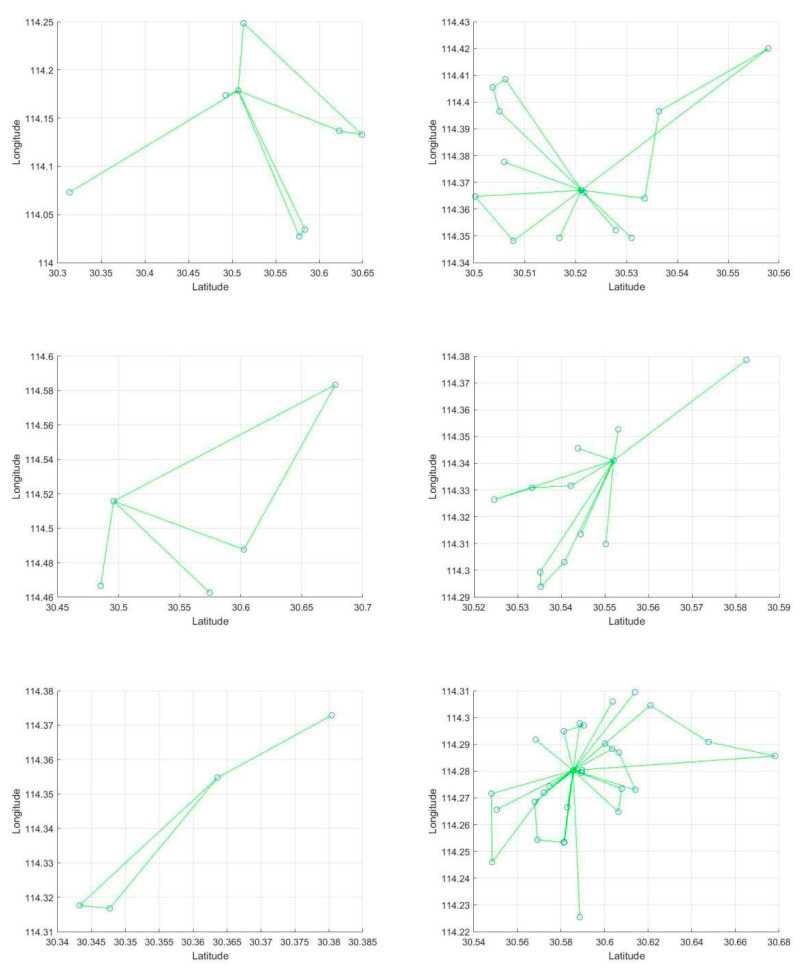
Vehicle allocation and paths for each transport station.

Focusing on each of the transport stations, we get the information shown in Figure 7.

In Figure 6 and Figure 7, the center point of each small area is the medical center of the area, and the green line segment indicates that if two points belong to the same subdivision area, they are connected by a line segment.

The distribution of cars is obviously different at disparate transport stations. In the areas where the hospitals are more concentrated, the routes are more complex.

**Figure 8:**
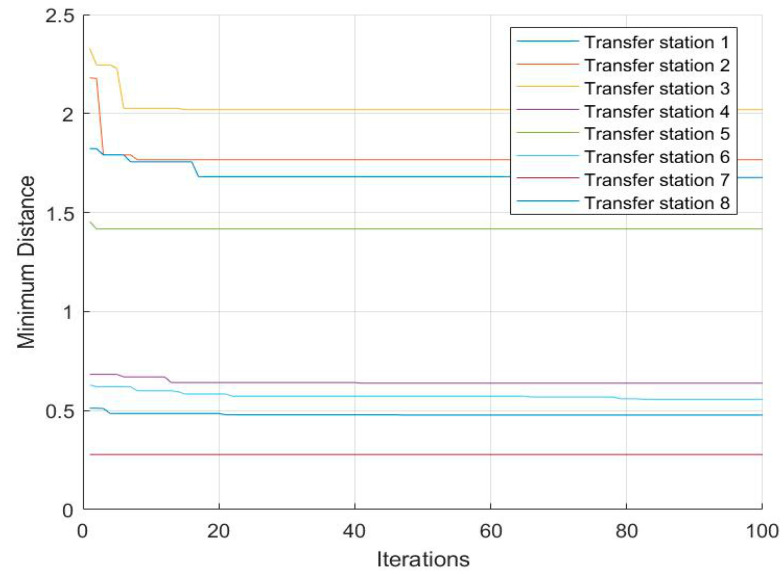
The convergence of each transit path with the increase of iteration times.

With the increase in the number of iterations, the convergence of the algorithm is shown in Figure 8.

As can be seen in Figure 8, with the increase of the number of iterations, the results gradually converge and obtain a relatively stable optimal solution.

In view of this, we are certain that the model of this paper has a good fit and dispatching effect even for Wuhan, which is a very complicated city. Therefore, by giving different parameters to different cities, it is quite convenient to apply our strategy, which would provide an effective scheme to optimize the scheduling of the whole urban transport system of medical waste.

In past studies, Balvinder Singh Gill et al. studied the transmission of COVID-19 in Malaysia [42], and Carol I. Blvd et al. made algorithmic scheduling for the delivery of medical forces [43]. Compared to their approach, our approach is more comprehensive, practical, and provides a better picture of what delivery scheduling actually looks like when COVID-19 outbreaks occur in cities.

## 5. Conclusions

The outbreak of COVID-19 in Wuhan has exposed the inefficiency of transporting medical waste and urges us to solve this impending problem.

Therefore, in this paper, several temporary storage points were discussed above, according to the environmental impacts and assessment criteria, utilizing the Q-value method to allocate medical waste transport vehicles, and applying the immune-based ant colony algorithm, together with the tabu search algorithm, to arrange the correct pathways of waste transportation. Eventually, a complete “build–match–transport” system model for medical waste is established during these procedures.

The application of this model to the epidemic situation of Wuhan has achieved excellent results, which has practical significance and enlightenment to the emergency response and dispatch of Wuhan and other major cities.

## Figures and Tables

**Table 1:** Exclusion criteria for areas not suitable for the construction of medical waste disposal stations.

Category	Exclusion Buffer
Drainage	1000 m
National Highway, Provincial HighwayUrban Express, Express	500 m
Green Space, Parks	1000 m
Towns, Schools	1000 m
Cultivated Land, Woodland, Grassland, Water Area	All Exclude

**Table 2:** The amount of medical waste generated by different grades of hospitals. Unit: kg/(per bed·per day).

Category	Classification Standard	Yield
Large Hospitals	With more than 300 beds	0.74
Provincial and Key Municipal Hospitals	Provincial Capital or Specifically City	0.6
City Hospitals	City	0.48

**Table 3:** Exclusion criteria for areas not suitable for the construction of medical waste disposal stations.

Relay Station Serial Number	Transit Station Latitude	Relay Accuracy
1	30.63143408	114.3805356
2	30.911936	114.367332
3	30.507047	114.178504
4	30.52111762	114.3670874
5	30.495884	114.515719
6	30.55204	114.341055
7	30.36358	114.354765
8	30.585537	114.280239

**Table 4:** Exclusion criteria for areas not suitable for the construction of medical waste disposal stations.

The Serial Numbers of the Transport Stations	Amount of Waste	*p*	*n*	ε
1	10.79	0.104747	5	0.004747
2	7.25	0.070382	4	0.009618
3	8.5	0.082516	4	0.002516
4	16.12	0.15649	8	0.00351
5	6.04	0.058635	3	0.001365
6	14.95	0.145132	7	0.005132
7	4.25	0.041258	2	0.001258
8	35.11	0.340841	17	0.000841

The cumulative error is ε=0.02897. This result is in line with the actual demand, which means the distribution effect is good.
